# Editorial: Mechanistic and statistical modeling approaches to study alloimmune T-cell responses

**DOI:** 10.3389/fimmu.2025.1591083

**Published:** 2025-04-16

**Authors:** Mario Castro, Josep Bassaganya-Riera, Miguel Fribourg

**Affiliations:** ^1^ Instituto de Investigación Tecnológica (IIT), Comillas Pontifical University, Madrid, Spain; ^2^ The NIMML Institute, Blacksburg, VA, United States; ^3^ Translational Transplant Research Center, Department of Medicine, Icahn School of Medicine at Mount Sinai, New York, NY, United States

**Keywords:** T cells, statistical methods, computational modeling, alloimmune, mechanism

Heart and kidney transplant graft survival is suboptimal, with an average half-life of approximately 10 years. T-cells play a crucial role in graft rejection. Following transplantation, antigen recognition of allo-peptides and HLA molecules leads to the activation of donor-reactive T-cells, which underlie potential T-cell-mediated rejection (TCMR) and can promote donor-specific antibody (DSA) formation. T-cell-targeting immunosuppressive therapies currently used in transplant recipients exert their effect on all T cells. The characteristics and the dynamics of T-cell alloimmune responses remain largely undefined, thus hindering our ability to target them selectively.

The advent of single-cell technologies and T-cell receptor (TCR) sequencing has constituted an inflection point in our ability to probe and monitor alloimmune responses. However, these come with the challenge of extracting relevant biological insight from the large datasets generated. Devising novel mechanistic mathematical and statistical modeling approaches is a promising strategy to address this unmet need.

The six papers in the “Mechanistic and Statistical Modeling Approaches to Study Alloimmune T-cell Responses” Research Topic explore critical aspects that shape T cell responses that can directly be applied to alloimmune responses in the context of transplantation and immunosuppressive therapies: heterogeneity of human CD8+ T cell responses to antigens, mathematical modeling of T cell recirculation in mice, insights into asymmetric T-cell division and its implications for immunotherapy, identification of diagnostic markers in the context of ischemia, the pre-organized landscape of T cell surfaces, and network analysis of immune repertoires. These studies collectively aim to enhance our understanding of T-cell dynamics and improve therapeutic strategies for transplant recipients.

Harris et al. propose a novel framework to quantify CD8+ T cell responses to vaccine antigens, considering human HLA heterogeneity and viral evolution. They introduce coverage metrics to assess vaccine-induced immune protection and characterize immuno-dominant epitopes. Their approach is demonstrated using the Ebola virus, SARS-CoV-2, and Burkholderia pseudomallei proteins. The study highlights the importance of accounting for genetic diversity and T cell receptor variability in vaccine design, aiming to improve predictions of immune outcomes and enhance vaccine efficacy.

Nikitich et al. developed a physiologically-based pharmacokinetic (PBPK) model to describe T cell homeostasis and the kinetics of exogenously administered T cells in mice. Their model integrates endogenous T cell turnover with pharmacokinetic aspects of engineered T cells, providing insights into T cell migration and distribution across various organs. The study highlights the importance of CCR7 expression in T cell trafficking. It offers a quantitative framework for predicting the distribution of adoptive T-cell therapies, aiming to enhance the development and optimization of such treatments.

Kaminskiy et al. provide a comprehensive overview of asymmetric T-cell division, emphasizing its significance in T-cell biology and immunotherapy. They explore the mechanisms governing asymmetric division in various T-cell subsets, highlighting the role of fate-determining factors and transcriptional and epigenetic regulation. The paper also delves into the interplay between T-cell receptor signaling and asymmetric division geometry, offering insights into the spatial organization and its impact on cellular fate. This work aims to guide researchers in selecting appropriate models to study asymmetric division in T cells.

Geng et al. identified and validated platelet-related diagnostic markers for ischemic stroke (IS) using bioinformatics and machine learning. They integrated datasets to find differentially expressed genes, isolating 51 platelet-related genes. Six key biomarkers (APP, THBS1, F13A1, SRC, PPBP, and VCL) were identified for a diagnostic model validated in rat models. Additionally, they screened potential antiplatelet drugs, highlighting alpha-linolenic acid and ciprofibrate for their efficacy in improving coagulation function and reducing cerebral infarction. This study advances early IS diagnosis and treatment strategies.

Jung reviews the pre-organized landscape of T cell surfaces, focusing on the spatial arrangement of key receptors and signalling molecules in their resting state. He highlights the role of super-resolution microscopy in revealing nanoscale clusters of T cell receptors (TCRs) and excluding CD45 from microvilli tips. The study emphasizes the importance of these pre-organized structures in optimizing T cell activation and signalling, providing insights into the molecular mechanisms that prepare T cells for rapid and effective immune responses.

Finally, Yang et al. developed the Network Analysis of Immune Repertoire (NAIR) to analyze T-cell receptor (TCR) sequences. Their approach uses advanced statistical methods to characterize TCR sequence changes and identify disease-specific clusters. By applying this to COVID-19 datasets, they identified TCRs associated with COVID-19 and validated their findings using the MIRA database. The study highlights the potential of NAIR to uncover disease-specific immune responses, providing a valuable tool for understanding adaptive immunity and improving disease diagnosis and monitoring.

The studies in this Research Topic, although not all focused directly on alloimmune responses, illustrate how statistical and mechanistic quantitative frameworks can help us understand how these T cell responses are mounted, organized, and evolve over time, thus helping accelerate the process of gleaning biological insight.

